# Phytochemical-based nanodrugs going beyond the state-of-the-art in cancer management—Targeting cancer stem cells in the framework of predictive, preventive, personalized medicine

**DOI:** 10.3389/fphar.2023.1121950

**Published:** 2023-03-23

**Authors:** Lenka Koklesova, Jana Jakubikova, Dana Cholujova, Marek Samec, Alena Mazurakova, Miroslava Šudomová, Martin Pec, Sherif T. S. Hassan, Kamil Biringer, Dietrich Büsselberg, Tatiana Hurtova, Olga Golubnitschaja, Peter Kubatka

**Affiliations:** ^1^ Clinic of Obstetrics and Gynecology, Jessenius Faculty of Medicine, Comenius University in Bratislava, Martin, Slovakia; ^2^ Cancer Research Institute, Department of Tumor Immunology, Biomedical Research Center, Slovak Academy of Sciences, Bratislava, Slovakia; ^3^ Centre for Advanced Material Application, Slovak Academy of Sciences, Bratislava, Slovakia; ^4^ Department of Pathological Physiology, Jessenius Faculty of Medicine, Comenius University in Bratislava, Martin, Slovakia; ^5^ Department of Medical Biology, Jessenius Faculty of Medicine, Comenius University in Bratislava, Martin, Slovakia; ^6^ Museum of Literature in Moravia, Rajhrad, Czech Republic; ^7^ Department of Applied Ecology, Faculty of Environmental Sciences, Czech University of Life Sciences Prague, Prague, Czech Republic; ^8^ Department of Physiology and Biophysics, Weill Cornell Medicine-Qatar, Education City, Qatar Foundation, Doha, Qatar; ^9^ Department of Dermatology, Comenius University in Bratislava, Jessenius Faculty of Medicine in Martin and University Hospital Martin, Martin, Slovakia; ^10^ Predictive, Preventive, Personalised (3P) Medicine, Department of Radiation Oncology, University Hospital Bonn, Rheinische Friedrich-Wilhelms-Universität Bonn, Bonn, Germany

**Keywords:** nanomedicine, nanoparticles, phytochemicals, plant-derived foods, cancer stem cells therapy, predictive preventive personalized medicine, primary secondary tertiary care

## Abstract

Cancer causes many deaths worldwide each year, especially due to tumor heterogeneity leading to disease progression and treatment failure. Targeted treatment of heterogeneous population of cells - cancer stem cells is still an issue in protecting affected individuals against associated multidrug resistance and disease progression. Nanotherapeutic agents have the potential to go beyond state-of-the-art approaches in overall cancer management. Specially assembled nanoparticles act as carriers for targeted drug delivery. Several nanodrugs have already been approved by the US Food and Drug Administration (FDA) for treating different cancer types. Phytochemicals isolated from plants demonstrate considerable potential for nanomedical applications in oncology thanks to their antioxidant, anti-inflammatory, anti-proliferative, and other health benefits. Phytochemical-based NPs can enhance anticancer therapeutic effects, improve cellular uptake of therapeutic agents, and mitigate the side effects of toxic anticancer treatments. Per evidence, phytochemical-based NPs can specifically target CSCs decreasing risks of tumor relapse and metastatic disease manifestation. Therefore, this review focuses on current outlook of phytochemical-based NPs and their potential targeting CSCs in cancer research studies and their consideration in the framework of predictive, preventive, and personalized medicine (3PM).

## 1 Introduction

Cancer is a leading cause of death worldwide. According to GLOBOCAN 2020, cancer estimated 19.3 million new cancer cases and almost 10.0 million cancer deaths in 2020 (Sung et al., 2021). In cancer, the genome instability and mutations are the reason of various changes in organism, including avoiding immune destruction, deregulation of cellular energetics, promotion of inflammation, sustaining proliferative signaling, evading growth suppressors, resisting cell death, enabling replicative immortality, activating angiogenesis, invasion and metastasis that consequently lead to disease progression (Hanahan and Weinberg, 2011). Moreover, disease progression and treatment failure are also commonly caused by tumor heterogeneity. There are two types of tumor heterogeneity: inter-tumor heterogeneity (between cancers from different patients) and intra-tumor heterogeneity (within a single tumor). The second one is characterized by phenotypic diversity through alterations in genetic or epigenetic abnormalities, apoptosis, tumor growth, and other hallmarks of cancer (Prasetyanti and Medema, 2017). Furthermore, many tumors contain a heterogeneous population of cells, including cancer stem cells (CSCs) that differentiate into cells to initiate tumor formation (Dick, 2008). CSCs also exert self-renewal and differentiation properties that often lead to the ineffectiveness of conventional therapy to eliminate CSCs. Consequently, the failure in therapy due to treatment resistance often causes tumor relapse and metastases (Lathia et al., 2020; Babaei et al., 2021). Treatment resistance or multi-drug resistance (MDR) describe the resistance to various unrelated therapies, including radiotherapy, chemotherapy, hypoxia, and immunotherapy. Besides, MDR occurs in up to 70% of cancers at the time of diagnosis (Riganti and Contino, 2019; Li et al., 2021a). Moreover, treatment resistance is not associated only with CSCs but MDR exerts multi-factorial character caused by epithelial-mesenchymal transition (EMT), acquired mutations, drug efflux through ABC transporters, drug efflux mediated by extracellular vesicles, drug-loaded lysosomes undergoing exocytosis, deregulation of key signaling pathways, deregulation of cell death mechanisms, activation of DNA damage response, and epigenetic alterations (Assaraf et al., 2019; Li et al., 2021a).Therefore, developing novel potential drugs to overcome the MDR of CSCs is crucial.

Plant-based foods are rich in various phytochemicals that exert many anticancer activities, including proapoptotic, anti-angiogenic, anti-metastatic, anti-inflammatory, antioxidant, or anti-genotoxic effects. However, the therapeutic efficacy can be low due to their low oral bioavailability and poor aqueous solubility (Koklesova et al., 2020a). On the other hand, an encapsulation of phytochemicals into nanocarriers can represent a potential drug delivery system in cancer management. Specific drug delivery into cancer cells and their release at the targeted site can enhance their antineoplastic properties (Kumar et al., 2022). Increased anticancer efficacy can be also achieved by combining various phytochemicals with conventional therapy or other NPs that can be activated through hyperthermia or photothermia (Sun et al., 2015; Li et al., 2019; Jose et al., 2020). Additionally, specifically designed phytochemical-based nanodrug can target CSCs and eliminate them, potentially reversing resistance to therapy or preventing migration and metastasis (Kuo et al., 2019; Yang et al., 2020; Gu et al., 2021).

Nanotechnology is widely used in different areas, including electronics, cosmetics, and diagnostic and therapeutic medical applications (Najahi-Missaoui et al., 2020). The field of nanotechnology in medicine, known as nanomedicine, has multiplied during the last few decades. Nanomedicine includes the use of nano-sized (1–1,000 nm) particles (NPs) as potential therapeutic drugs for various diseases (Missaoui et al., 2018; Tabassum et al., 2018). In cancer research, specifically designed NPs with various sizes and properties represent a new way of delivery systems to targeted delivery into tumor sites without harming the surrounding healthy tissues (Patra et al., 2018). Therefore, this review focuses on the current outlook on phytochemical-based nanodrugs and their potential targeting CSCs in cancer research studies.

## 2 Nanoparticles

**FIGURE 1:**
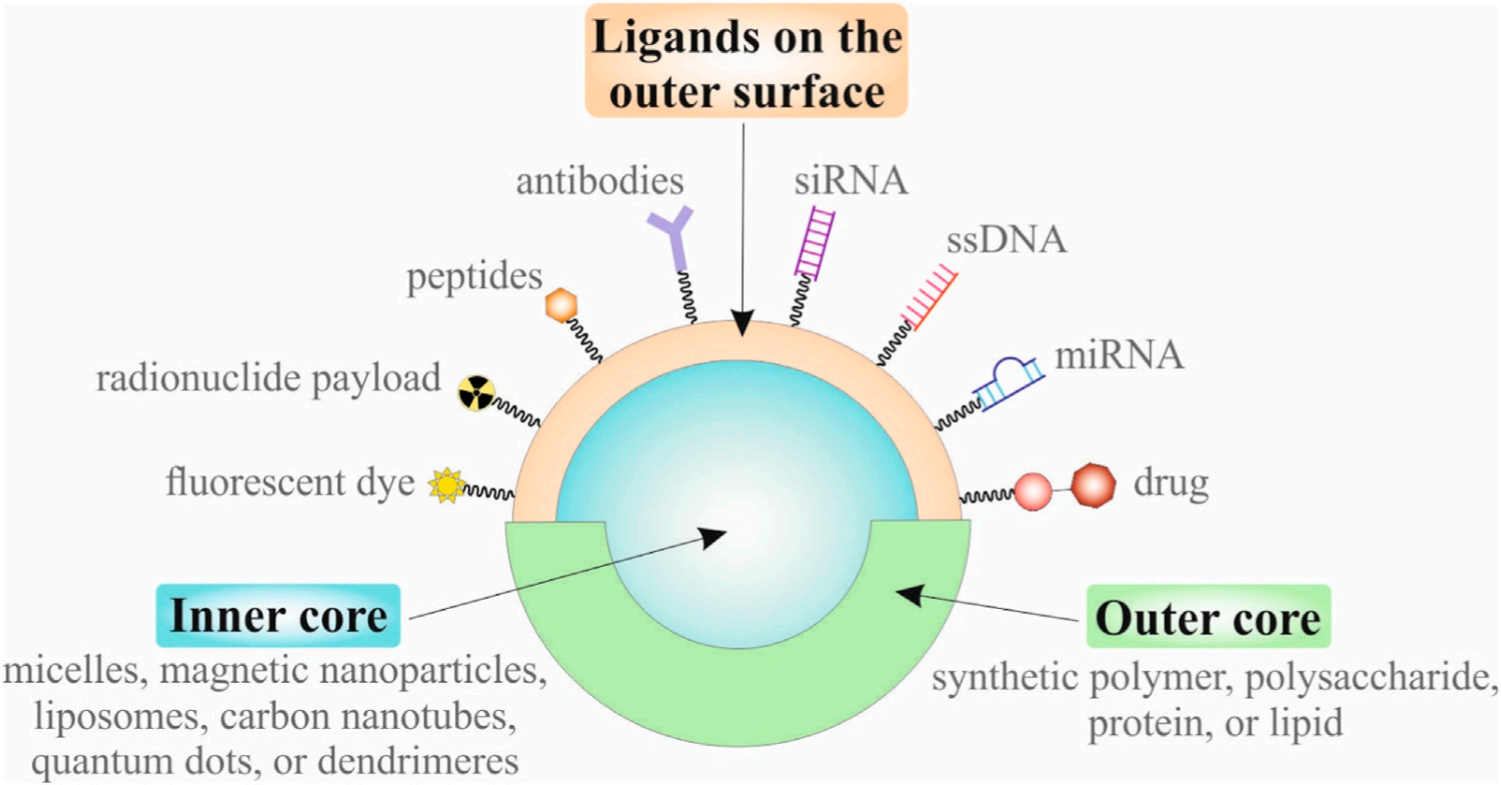
Schematic structure of the multifunctional nanoparticle.

Nanomedicine is represented by small-sized (nanoscale, 1–1,000 nm) drug delivery systems that specifically deliver drug molecules to pathologic sites and accumulate at the target site (Gwinn and Vallyathan, 2006; Tabassum et al., 2018). NPs can also have various shapes, including spherical, rod, oval, cubic, triangular, star, needle, octahedral, flower, cluster, cylinder, branched, platelets, hexagonal, pentagonal, and others (Hamida et al., 2020). NPs can be divided into six groups according to their composition of inner and outer core (Najahi-Missaoui et al., 2020). Moreover, the surface of NPs consists of various ligands with the ability to target damaged (e.g., cancer) cells thanks to their specific selective binding to the overexpressed receptors (Sun et al., 2014). Furthermore, NPs are commonly coated by various agents for better biocompatibility and biodegradability (Singh, 2010). Figure 1 illustrates the schematic structure of NP consisting of an inner core, outer core, and ligands on the outer surface.

**FIGURE 2:**
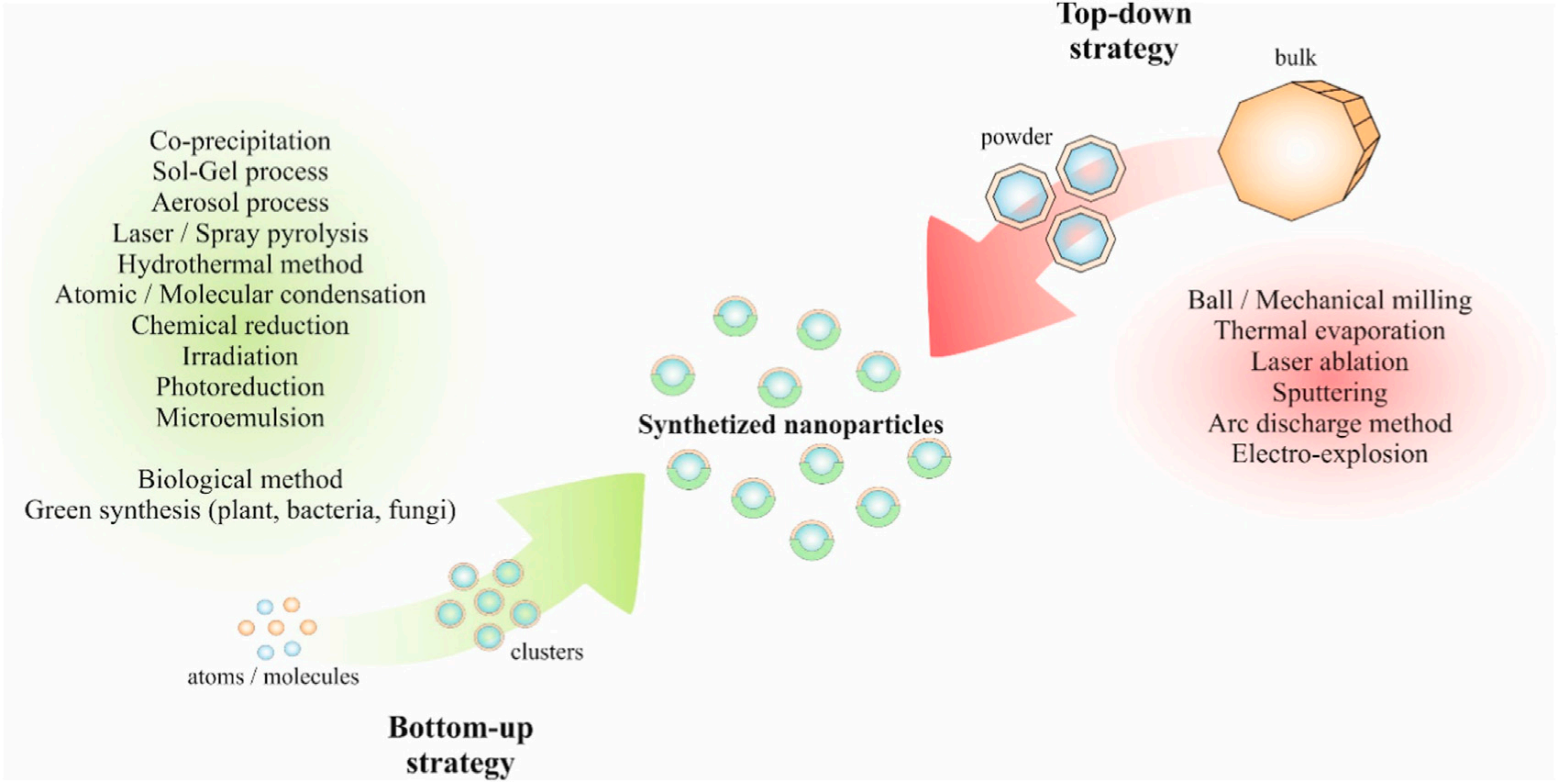
Synthesis of NPs by bottom-up or top-down strategies.

NPs can be synthesized in two ways: by bottom-up strategy or top-down strategies, as illustrated in Figure 2. A bottom-up strategy is based on nucleating atomic-sized materials into the eventual NPs. The top-down strategy represents physical degradation of bulk material producing smaller molecules and NPs (Nagarajan, 2008).

## 3 Tumor cells targeting by nanoparticles

Nanosize of NPs can overcome various biological barriers within the body, such as entering the cell and various cellular compartments (nucleus) (De Jong and Borm, 2008). Therefore, several NPs exhibit the potential for their clinical application; however, their usability depends on various factors such as size, shape, surface functionality, low or no toxicity of the nanocarrier, physical and chemical properties, solubility, stability, drug loading efficiency, drug release, and potential distribution to different organs (Gwinn and Vallyathan, 2006; Puri et al., 2009; Singh, 2010). NPs can act as tumor detector that detects a diseased/cancer site where it accumulates and specifically triggers the therapeutic activity of a circulating drug carrier. Specific targeting of NPs into cancer cells is determined predominantly by ligands on their surface (Zwicke et al., 2012; Yang et al., 2022). At cancer sites, several nanodrugs act as DNA-damaging, immunostimulant, microtubule-inhibiting, or hormone agonist agents that trigger various anticancer pathways (FramptonMifamurtide, 2010; Barenholz, 2012; Gawde et al., 2018; Fu et al., 2020). In hyperthermia events (to 40°C–45°C), cells are susceptible to various forms of damage. Hyperthermia activates various immunological responses, enhances tumor blood flow and oxygenation through higher permeability and vascular perfusion, decreases oxygen consumption, and increases tissue oxygenation by a shift toward anaerobic metabolism. Every mentioned mechanism leads to the alteration of the extracellular microenvironment (Chatterjee et al., 2011). Cancer cells are more thermosensitive than normal healthy cells. Various types of nanostructure can be used for hyperthermia activity, including silica-gold and gold nanoshells, gold nanorods, core-shell gold NPs, solid gold NPs, and carbon nanotubes (Cherukuri et al., 2010; Kaur et al., 2016). One of other hyperthermia event, magnetic hyperthermia can convert the magnetic energy of magnetic NPs into heat energy in the magnetic field. Therefore, magnetic NPs (e.g., metal NPs) can target and kill cancer cells with low toxicity to normal cells. Moreover, a combination of NPs-based magnetic hyperthermia therapy and radiotherapy or chemotherapy can achieve higher thermosensitivity of cancer cells (Maeda, 2001; Jose et al., 2020). Photothermal therapy represents a minimally invasive procedure for cancer treatment. Photo-induced hyperthermia that converts light to heat can be achieved by pulsed and continuous waves or pulsed near-infrared laser irradiation in appropriate dosage (Sahu et al., 2018). For example, gold nanostars presented by star-shaped geometry show therapeutic potential in cancer. Their shape increases light absorption leading to high photon/light-to-heat conversion efficiency through the plasmonic effect. Subsequently, increased temperature causes cell damage at the tumor site (Liu et al., 2018a).

### 3.1 Nanodrugs in cancer therapy

The US Food and Drug Administration (FDA) approved several nanodrugs for treating various cancer types. FDA-approved nanodrugs used in cancer therapy have different specific targets (e.g., DNA damage, immunostimulation, microtubule, protein synthesis, or hormone inhibition) or formulations. Some of them consist of metallic NPs (Aurimmune®, AuNPs®), polymer-drug conjugates (Eligard®, SMANCS), lipid-based nanoformulations (Marqibo®, Doxil®), recombinant virus (Gendicine®), drug targeted antibody (Kadcyla®), or herbal NPs (nanoformulated curcumin) (Alphandéry et al., 2015). In 1995, the first FDA-approved nanodrug was Doxil®, polyethylene glycol (PEG)ylated liposomal doxorubicin, indicated for the treatment of metastatic ovarian cancer and AIDS-related Kaposi’s sarcoma (Barenholz, 2012). Table 1 represents an overview of some FDA-approved nanodrugs used in cancer therapies.

**TABLE 1:** An overview of FDA-approved nanodrugs used in cancer therapies.

Nanodrug	Nanoformulation	Cancer therapy	Mechanism of action	References
Abraxane®	Nab-paclitaxel	Advanced metastatic breast, lung, or pancreatic cancer	Antimicrotubule agent	Gawde et al. (2018)
AuNPs	PEGylated gold NPs conjugated with anti-EGFR antibodies	EGFR-overexpressing tumors (e.g., head and neck squamous cell carcinomas), and other solid tumors	Targeting cells by coating with anti-EGFR monoclonal drug antibodies	Zhang et al. (2017), Liszbinski et al. (2020)
Aurimune®	PEGylated TNF-α coated gold nanospheres	Solid tumors	Immunostimulants, photothermally-activated physical and biological effects	Libutti et al. (2010)
Doxil®	PEGylated liposomal doxorubicin	Metastatic ovarian cancer and AIDS-related Kaposi’s sarcoma	DNA damaging/synthesis inhibitor	Barenholz (2012)
Eligard®	PEGylated leuprolide acetate	Advanced prostate cancer	A gonadotropin-releasing hormone agonist	Fu et al. (2020)
Gendicine®	Recombinant human p53 adenovirus	Head and neck squamous cell carcinoma	Gene therapy for cancer patients with mutated p53 genes	Peng (2005)
Kadcyla®	Trastuzumab emtansine	Early HER2+ breast cancer	Anti-HER2 monoclonal antibody	von Minckwitz et al. (2019)
Marqibo®	Non-PEGylated liposomal vincristine	Philadelphia chromosome-negative acute lymphoblastic leukemia, Hodgkin and Non-Hodgkin lymphoma, or lymphoid blast crisis of chronic myeloid leukemia	Microtubules inhibitor	Below and M Das (2022)
MEPACT	Liposomal muramyl tripeptide phosphatidyl ethanolamine	Non-metastatic osteosarcoma	Immunomodulator, activates monocytes, TNF-α, IL-1b, IL-6, IL-8, and IL-12, and macrophages	FramptonMifamurtide (2010)
MM302	HER2-targeted PEGylated antibody–liposomal doxorubicin	Advanced HER2-positive breast cancers	DNA damaging/synthesis inhibitor	Martin and López-Tarruella (2016)
Nanotherm®	Iron oxide NPs coated with amino-silane	Glioblastoma	Magnetic hyperthermia therapy	Mahmoudi et al. (2018)
Onivyde^®^	PEGylated liposomal irinotecan	Metastatic pancreatic ductal adenocarcinoma	DNA damaging, Single-strand breaks induction, the release of torsional strain by topoisomerase 1	Frampton (2020)
SMANCS	Styrene-maleic acid copolymer-conjugated neocarzinostatin	Advanced and recurrent hepatocellular carcinoma	DNA damaging/synthesis inhibitor	Abe and Otsuki (2002)

**Abbreviations:** PEG, polyethylene glycol; nab, nanoparticle albumin-bound, TNF-α, tumor necrosis factor alpha; IL, interleukin; AIDS, acquired immune deficiency syndrome; EGFR, epidermal growth factor receptor; NPs, nanoparticles.

In addition to the above-mentioned mechanisms of action, the accumulation of NPs at the diseased site causes mitochondria damage and dysfunction, upregulation of apoptotic factors, DNA fragmentation, membrane damage of cancer cells, oxidation of enzymes and proteins, protein denaturation, disassembly of ribosomes, generation of reactive oxygen species (ROS), interruption of electron transport (Roy et al., 2019; Barabadi et al., 2020; Chaudhary et al., 2020). Moreover, the accumulation of NPs at the diseased site demonstrates the diagnostic potential because NPs can act as potential contrast agents for X-ray (gold NPs), magnetic resonance imaging (MRI) (magnetic NPs), computed tomography (CT) and MRI (hybrid NPs from iron oxide and gold) (Smith et al., 2012). Various NPs have been evaluated as potential contrast agents in cancer diagnostics; however, their clinical applications are limited, especially due to their insufficient assessment of biodegradation, elimination and toxicity (Baetke et al., 2015).

Furthermore, thanks to recent FDA approvals of lipid NP-loaded mRNA vaccines for the prevention of COVID-19, the lipid NP-based mRNA vaccines could represent promising way also in cancer therapy in near future (Miao et al., 2021). For example, lipid NP-based mRNA vaccine known as BI1361849 (CV9202) combined with local radiation evaluated in Ib clinical trial (NCT01915524) in patients (n = 26) with stage IV of non-small cell lung cancer. In the majority of patients, the vaccine increased CD4+ and/or CD8+ T cells and BI1361849 antigen-specific immune responses (Papachristofilou et al., 2019). Similarly, enhanced immune responses in patients with stage IIIB/IV non-small cell lung cancer were observed after vaccine BI1361849 in combination with a checkpoint inhibitor, anti-CTLA-4 (tremlimumab) and anti-PD-L1 (duvalumab) in phase I/II study (NCT03164772) (Sebastian et al., 2019). In this way, other mRNA vaccines based on lipid NPs revealed potential in cancer immunotherapy of solid tumors (Huang et al., 2022).

### 3.2 Phytochemical-based nanodrugs

**FIGURE 3:**
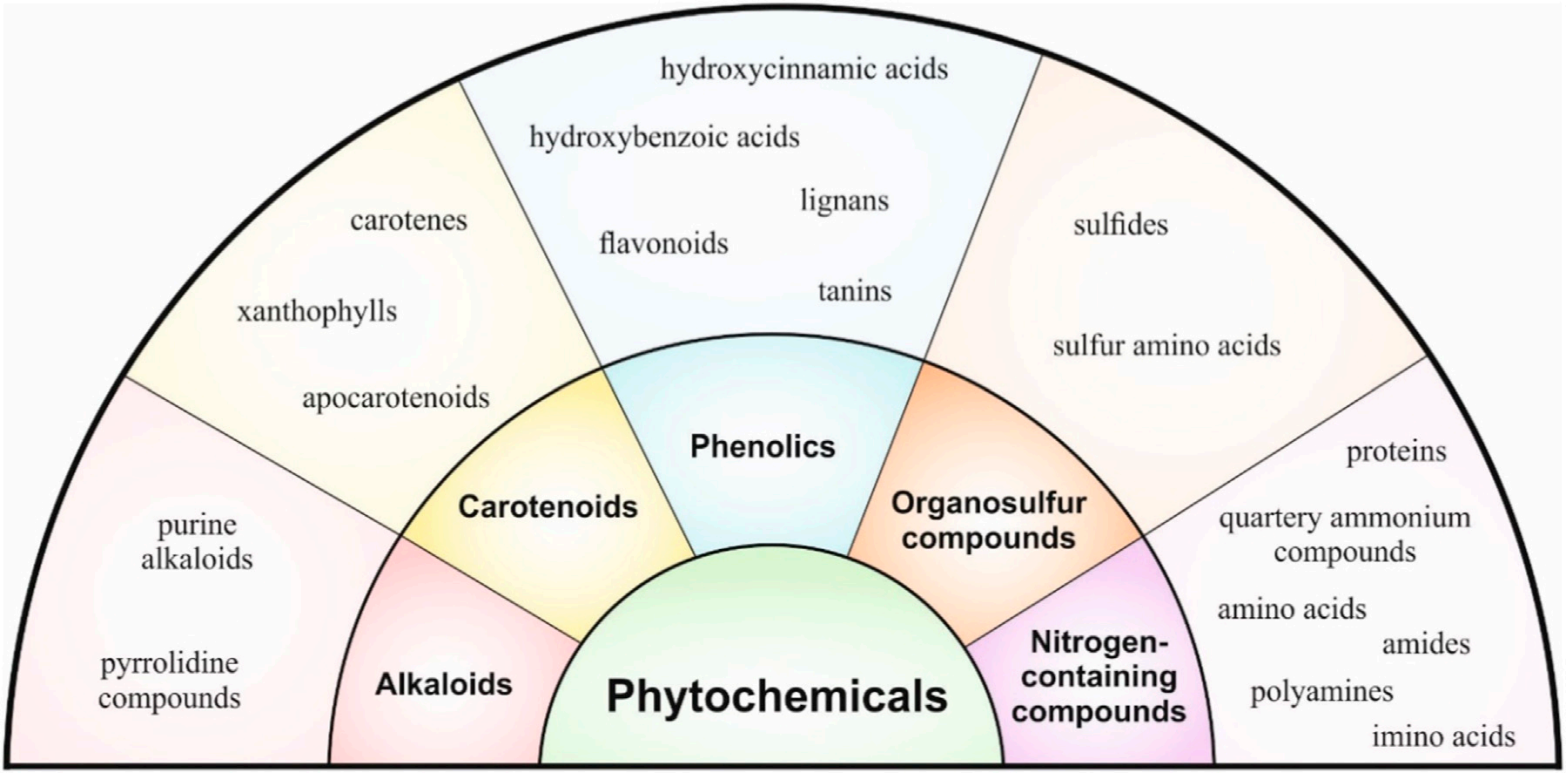
Classification of phytochemicals.

Phytochemicals are biologically active compounds commonly found in plant-based food such as fruits, vegetables, grains, or nuts, exerting anticancer, antioxidant, anti-inflammatory, immunomodulatory, and other beneficial properties (Cencic and Chingwaru, 2010; Koklesova et al., 2020b). Phytochemicals are classified into five basic groups: phenolics, carotenoids, alkaloids, organosulfur, and nitrogen-containing compounds (Liu, 2004). Figure 3 describes the classification of phytochemicals into basic groups and subgroups.

Several preclinical and clinical studies demonstrate the anticancer potential of phytochemicals alone or their combination or combination with other drugs in preventive and therapeutic cancer management (Abotaleb et al., 2018; Koklesova et al., 2020b; Samec et al., 2020). Therefore, phytochemicals are suitable for nanomedicine, specifically for conjugating with various NPs or for encapsulation into nanocarriers. These nanophyto-formulations demonstrate various potential health benefits in infectious, cardiovascular, and neurodegenerative diseases as well as cancer (Nazer et al., 2020; Hesari et al., 2021; Bhattacharya et al., 2022; Melim et al., 2022).

Despite several FDA-approved nanodrugs for cancer therapy, for medical progress is still important to develop novel drugs or their alterations that could be more sensitive and effective with less side effects or specifically stratified for patients. After all, the aim of nanotechnology is enhancing the bioavailability, solubility, absorption, and controlled-release of drugs (Patra et al., 2018). Natural products represent the low cost, low resistance, less toxic, and effective compounds (Dhupal and Chowdhury, 2020). Moreover, phytochemical-based nanodrugs can overcome the chemotherapeutic resistance of CSCs or can resensitize them to therapy (Chan et al., 2018; Shen et al., 2021).

### 3.3 Phytochemical-based nanodrugs in cancer research

Various preclinical and clinical studies focused on the phytochemicals conjugated NPs, especially in cancer research.

#### 3.3.1 Gold NPs

Resveratrol-conjugated gold nanoparticles (Res-AuNPs) exerted synergistic anti-tumor effects in human breast, pancreatic, and prostate cancer cells. 3× Res-AuNPs and 3× Res-GA-AuNPs revealed cytotoxic effects, enhanced bioavalability and cellular uptake when compared with 1× Res-AuNPs and 1× Res-GA-AuNPs. In conclusion, Res-AuNPs enhanced phytochemical drug carrier capabilities as a potential application for cancer therapy (Thipe et al., 2019). Furthermore, Res-AuNPs and Resveratrol-nanoemulsion inhibited the growth of BxPC-3pancreatic cancer cells and altered cell cycle regulation and apoptotic events (Inbaraj et al., 2021).

Multifunctional and spherical 20 nm AuNPs conjugated with withanolide-A, a phytocompound from *Withania somnifera*, demonstrated higher antiproliferative effects when compared with withanolide-A alone in the SKBR-3 breast cancer cell line (Tabassam et al., 2020).

Mango peel phytochemicals coated AuNPs and mangiferin, the most abundant phytochemical in mango peel, conjugated AuNPs were combined with plant phytochemicals from Amalaki (*Emblica officinalis*), Amra (*Mangifera indica*), Haridra (*Curcumin longa*), Babbula (*Acacia nilotica*), Yashtimadhu (*Glycyrrhiza glabra*) to create Nano Swarna Bhasma (NSB) drug. NSB drug revealed selective toxicity to MDA-MB-231 cancer cells, reduced tumor volume in MDA-MB-231 mice xenografts. Moreover, in a pilot clinical study, breast cancer patients demonstrated a partial response to the treatment without any disease progression. In summary, NSB therapy in patients with metastatic breast cancer exerted clinical benefits (Khoobchandani et al., 2020).

Silibinin-conjugated gold nanoparticles (Sb-AuNPs) effectively induced *in vitro* cell death against A549 lung cancer cells with long-term stability. The results showed that the efficacy of Sb improved 4–5 times in inhibiting the cancer cells after the conjugation with AuNPs (Ravi et al., 2022).

#### 3.3.2 Solid lipid NPs

The combination of curcumin and resveratrol solid lipid nanoparticles (Cur-Res-SLNs) inhibited cell migration of B16F10 melanoma cells. Moreover, Cur-Res-SLNs or Cur-Res solution (3:1) revealed strong synergism through the cell proliferation inhibition of SK-MEL-28 melanoma cells (Palliyage et al., 2021).

Moreover, erlotinib and quercetin-loaded solid lipid NPs (EQNPs) showed anticancer effects through increased cellular uptake of NPs.Moreover, EQNPs sensitized and enhanced the induction of apoptosis in Ertb-resistant A549/ER cells (Ganthala et al., 2022).

#### 3.3.3 Chitosan NPs

Quercetin encapsulated chitosan functionalized copper oxide nanoparticle (CuO-ChNPs-Q) demonstrated potent anticancer activity *in vitro* and *in vivo*. CuO-ChNPs-Q demonstrated cytotoxic effect against liver, breast, and colorectal cancer cells but safety of CuO-ChNPs-Q on WI38 human normal lung fibroblasts. In *vivo* study, CuO-ChNPs-Q reduced the breast tumor volume and proliferation, arrested the cell cycle, and induced apoptosis in DMBA-induced female rats (Elsayed et al., 2021).

In another investigation, a hydrogel nanocomposite of chitosan, halloysite, and graphitic-carbon nitride (Ch-HNT-gC3N4) was prepared and loaded by quercetin using an emulsification process to achieve quercetin sustained-release. The prepared drug-loaded delivery system exhibited excellent encapsulation and loading effectiveness, cytotoxic effect, and enhanced apoptotic activity in MCF-7 breast cancer cells (Sabzini et al., 2022).

In a combined *in vitro* and *in vivo* experiment, Zhou and others (2022) developed a new nanocarrier called chitosan-gelatin-epigallocatechin-3-gallate (Ch-G-EGCG) for systemic si‐TMEM44‐AS1 delivery that can silence TMEM44‐AS1 gene expression in gastric cancer cells and boost 5‐FU sensitivity in gastric cancer cells (Zhou et al., 2022).

#### 3.3.4 Poly (lactic-co-glycolic acid) NPs

In A549 and H1299 lung cancer cells, poly (lactic-co-glycolic acid) NPs loaded with epigallocatechin-3-gallate (PLGA-EGCG) demonstrated antiproliferative andapoptotic events. Furthermore, PLGA-EGCG-NPs decreased tumor volume and weight in the patient-derived xenograft model (Zhang et al., 2020).

Another study revealed that galactose-tailored poly (lactic-co-glycolic acid) NPs loaded with apigenin (API-GAL-NPs) exerted higher cellular internalization, cytotoxic and apoptotic effects in HepG2 human liver hepatocellular carcinoma cancer cells. In the diethylnitrosamine-induced hepatocellular carcinoma rat model, API-GAL-NPs reduced nodule formation and expression of matrix metalloproteinases and triggered apoptosis in the liver (Ganguly et al., 2021).

#### 3.3.5 Iron NPs

Another research group fabricated quercetin-ferrum nanoparticles (Q-F NPs) to improve photothermal therapy (PTT) by modulating the tumor immunosuppressive microenvironment. The prepared nano-photosensitizer induced cancer cell destruction and tumor antigen release, which in turn, stimulated dendritic cell maturation and T-cell activation. Furthermore, the Q-F NPs-PTT-treated mice displayed notably extended survival time and potent anti-tumor immune memory to control tumor metastasis and recurrence (Li et al., 2022).

#### 3.3.6 Folic acid and bovine serum albumin NPs

Difluorinated curcumin (CDF), a synthetic curcumin analog, encapsulated in folic acid and bovine serum albumin NPs (FA-BSA-CDF) and paclitaxel (PTX) encapsulated in folic acid and bovine serum albumin (FA-BSA-PTX) showed anticancer effect through targeting folate receptor and induction of apoptosis in folate overexpressing ovarian and cervical cancers. Separately treatment with either FA-BSA-PTX or FA-BSA-CDF decreased cell viability of SKOV-3 ovarian cancer and HeLa cervical cancer cells Furthermore, the combination of FA-BSA-PTX and FA-BSA-CDF revealed synergism and enhanced cancer cell-killing effect (Gawde et al., 2018).

#### 3.3.7 Zinc oxide NPs

Another *in vitro* investigation presented quercetin-functionalized wurtzite-type zinc oxide (ZnO-Q) NPs with potent anticancer action against human ovarian cancer cells by inducing intercellular oxidative stress and depolarization of the mitochondrial membrane. Besides, the prepared formulation generated late apoptosis *via* activating the intrinsic apoptosis signaling pathway in PA-1 cells (Ramalingam et al., 2022).

#### 3.3.8 Silica NPs

Resveratrol encapsulation into mesoporous silica nanoparticles (Res-MSNs) promoted its amorphization and enhanced drug release. Moreover, Res-MSNs reduced cell viability of human A375 and MNT-1 melanoma cells; however, with higher sensitivity in the amelanotic A375 cell line (Marinheiro et al., 2021).

#### 3.3.9 Poly (Glycerol Sebacate) NPs

*In vitro* study, curcumin-loaded nanoparticles of Poly (Glycerol Sebacate) (Cur-PGS-NPs) demonstrated cytotoxicity, altered cell cycle, and triggered apoptosis in human cervical cancer cells (Massironi et al., 2022).

#### 3.3.10 Micelles

Curcumin encapsulated into monomethyl PEG-polylactide (Cur-MPEG-PLA) micelles demonstrated anticancer potential for melanoma treatment *in vitro* and *in vivo*. Cur-MPEG-PLA micelles inhibited proliferation, induced apoptosis, and enhanced cellular uptake in B16 and A375 melanoma cells. Moreover, in mice bearing B16 or A375 subcutaneous melanoma, treatment by Cur-MPEG-PLA micelles decreased tumor volumes and inhibited neovascularization in tumor tissues (Wang et al., 2017).

At the nanoscale, dual-targeted diosmin and berberine hydrochloride-loaded casein micelles (DSN/BRB-CAS MCs) revealed cytotoxicity in HepG2 cells and hepatocellular carcinoma-bearing mice. These micelles decreased cell necrosis, inhibited tumor proliferation, angiogenesis, inflammation, and induced apoptosis (Abdelmoneem et al., 2018).

#### 3.3.11 Quantum dots

A phytochemical from some cruciferous vegetables called allyl isothiocyanate conjugated with silicon quantum dots (AITC- SiQDs) decreased cell viability in Caco-2 cells Moreover, AITC-SiQDs treatment caused a significant increase in ROS, induced DNA damage, and inhibited cell migration and tube formation in the 3D (HUVECs and MII perivascular cells) co-culture model (Liu et al., 2018b).

#### 3.3.12 Green-synthetized NPs and carrier-free NPs

The green-synthesized selenium NPs using apigenin (SeNPs-API) reduced cell proliferation and viability in MCF-7 breast cancer cells. Moreover, the treatment with SeNPs-API increased oxidative stress and ROS production, and triggered apoptosis through modulation of pro-apoptotic and anti-apoptotic markers (Al-Otaibi et al., 2022).

Carrier-free nanodrug (ASP-UA NPs) based on hydrophobic interactions consisting of ursolic acid, a pentacyclic triterpenoid, and aspirin, a non-steroidal anti-inflammatory drug, demonstrated anticancer effects. ASP-UA NPs significantly decreased cell viability in melanoma, cervical, liver, and breast cancer cells. *In vivo* metastasis assay revealed that ASP-UA NPs inhibited lung metastasis in mice injected with H22 hepatocellular carcinoma mouse cells (Li et al., 2018).

Table 2 describes the detailed anticancer effects of above-mentioned phytochemical-based nanodrugs. Interestingly, more than 300 clinical studies focused on nanotherapy in cancer research (clinicaltrial.gov); however, there is a lack of studies explicitly focused on phytochemical-based nanodrugs.

**TABLE 2:** Anticancer effects of phytochemical-based nanodrugs.

Phytochemical-based NPs	NPs size	NPs synthesis	Study details	Anticancer efficacy	References
Res-AuNPs	Res-AuNPs (56.1 nm), Res-GA-AuNPs (64.1 nm), 3 × Res-AuNPs(107.7 nm), 3 × Res-GA-AuNPs (187.7 nm)	Resveratrol reduced Au^3+^ to Au^0^ for the synthesis of Res-AuNPs, and gum arabic was used for further encapsulation of the NP surface	MDA-MB-231 human breast, PANC-1 pancreatic, and PC-3 prostate cancer cells	24-h incubation with Res-AuNPs at 42 μg/mL: ↑ cellular internalization, ↑ drug carrier capabilities, ↑ bioavailability, ↓ cell viability	Thipe et al. (2019)
Res-MSNs	Spheroidal (∼60 nm) MSNs	Synthesis based on (an aqueous) biphasic system	Human A375 and MNT-1 melanoma cells	↑ Res amorphization, ↑ drug release, ↓ cell viability	Marinheiro et al. (2021)
Cur-Res-SLNs	180.2 ± 7.7 nm in NPs diameter	High-shear homogenization method	B16F10 and SK-MEL-28 melanoma cells	↓ Cell migration, strong synergism, ↓ cell proliferation, higher drug release of Res compared to Cur, ↑ encapsulation efficiency and skin binding	Palliyage et al. (2021)
Res-AuNPs and Res-nanoemulsion	Mean particle size of Res-AuNPs (20.8 and 11.9 nm) and Res-nanoemulsion (14.1 nm)	Res-AuNPs were prepared by heating and stirring the mixture until the solution color turned red, Res-nanoemulsion prepared by sonication of the mixture	BxPC-3 pancreatic cancer cells	↓ Growth of BxPC-3 cells, modified cell cycle regulation, ↓ cyclin A, ↓ cyclin B, ↓ CDK1, ↓ CDK2, ↑ apoptosis, ↑ p53, ↑ p21, ↑ cytochrome c release, ↑ Bax, ↑ caspase-8, ↑ caspase-9, ↑ caspase-3, ↓ Bcl-2, ↑ cellular uptake	Inbaraj et al. (2021)
Cur-MPEG-PLA	Spherical Cur-MPEG-PLA micelles (34.5 nm)	Micelles synthesized by a single-step precipitation method	Murine B16 and human A375 melanoma cells; mice bearing B16 or A375 subcutaneous melanoma	*In vitro*: ↓ proliferation, ↓ Ki67, ↑ apoptosis, ↑ cellular uptake;	Wang et al. (2017)
				*In vivo*: ↓ tumor volumes, ↓ neovascularization, ↓ FITC–dextran uptake	
FA-BSA-CDF and FA-BSA-PTX NPs	FA-BSA-CDF (197.8 nm) and the FA-BSA-PTX (194.4 nm)	NPs prepared by desolvation technique based on a reported coacervation process	SKOV-3 ovarian cancer and HeLa cervical cancer cells	Targeting folate receptor, ↑ apoptosis	Gawde et al. (2018)
				Separately treatment: ↓ cell viability	
				Combination treatment: synergism and enhanced cancer cell killing effect	
Cur-loaded PGS-NPs	Average size of PGS NPs: 121 ± 11 nm - 124 ± 13 nm	Curcumin-loaded PGS-NPs prepared by nanoprecipitation	Human HPV18+ and HeLa cervical cancer cells	↑ cytotoxicity, ↑ apoptosis, ↑ p53, ↑ p21, ↑ Bax, ↓ viral HPV E6 oncogene, ↑ caspase-3, ↑ PARP, cell cycle arrest	Massironi et al. (2022)
AuNPs + withanolide-A	29.73 ± 0.650 nm	Chemical synthesis of withanolide-A 10 μg/mL with spherical 20 nm AuNP solution by Turkevich method	SKBR-3 breast cancer cells	↑ Antiproliferative effects, ↓ cell growth, ↑ cellular uptake↓ cell viability at the concentration of 40 μg/mL: AuNPs + withanolide-A (30%), withanolide-A alone (45%)	Tabassam et al. (2020)
*Nano Swarna Bhasma* drug	Core size (35 ± 2 nm), hydrodynamic size of MGF-AuNPs (55 ± 5 nm), and hydrodynamic size of MP-AuNPs (65 ± 5 nm)	NPs synthesized by redox reactions - electrons from phytochemicals reduced gold salt to the corresponding AuNPs	Preclinical study: MDA-MB-231 breast cancer cells and HAECs human aortic endothelial cells, SCID female mice were inoculated with MDA-MB-231 cells	↑ anti‐inflammatory, ↑ anticancer, ↑ antioxidant activities, ↑ selectively toxicity of cancer cells, ↓ toxicity of normal cells, ↓ tumor volume	Khoobchandani et al. (2020)
			Clinical pilot study: patients with breast cancer - Arm A (standard of Care drugs) (n = 3), Arm B (standard of care treatment along with the NSB drug) (n = 3) for 12 weeks	Clinical benefits, partial response to treatment, no progression of disease, mild severity of adverse events	
AITC- SiQDs	From 11.85 ± 0.05 to 22.70 ± 0.50 nm	NPs synthesized by galvanostatic anodization of porous silicon layer	HUVECs, HepG2 hepatocellular carcinoma, murine MII perivascular, Caco-2 colorectal adenocarcinoma cells	↓ Cell viability, ↑ ROS, ↑ Nrf2 translocation into nucleus, ↓ cell migration, ↓ tube formation higher dose: ↑ DNA damage, ↓ DNA repair protein Ku70	Liu et al. (2018b)
BRB/DSN-CAS MCs	CAS-MCs (186.7–295.4 nm), BRB/DSN-CAS MCs (253.1 ± 0.38 nm)	Micelles are prepared by stirring in methanolic solution to the resultant micellar dispersion	Mice with hepatocellular carcinoma, HepG2 liver cancer cells	↓ NF-κB, ↓ TNF-α, ↓ tumor proliferation, ↓ Ki67, ↓ angiogenesis, ↓ VEGF, ↓ inflammation, ↓ COX-2, ↑ apoptosis, ↑ caspase-3	Abdelmoneem et al. (2018)
Asp-UA NPs	Asp-UA NPs in methanol 231.1 nm (200 μM), 186.4 nm (100 μM), 101.7 nm (50 μM)	Chemical and ultrasound synthesis of Asp-UA NPs	B16F10 melanoma, HeLa cervical, HepG2 liver, and MCF7 breast cancer cell lines	↓ cell viability, ↓ metastasis, ↓ cancer nodules on lung surfaces, ↑ cellular uptake	Li et al. (2018)
CuO-ChNPs-Q	Spherical CuONPs with a size 26 ± 3 nm and CuO-ChNPs-Q with size about 50 ± 3 nm	CuONPs prepared by precipitation method using copper nitrate (Cu(NO3)2) and copper chloride (CuCl2), Q solution gradually added to functionalized CuONPs during stirring with magnetic starrier to CuO-ChNPs-Q preparation	HepG-2 liver, MCF-7 breast, and CaCo-2 colorectal cancer human cell lines and WI38 human normal lung fibroblasts; DMBA-induced mammary carcinoma in female Sprague-Dawley rats	*In vitro*: ↑ cytotoxic effect incancer cells, safety in WI38 normal cells	Elsayed et al. (2021)
				*In vivo:* ↓ breast tumor weight and volume, ↓ proliferation, ↓ PCNA gene, ↑ apoptosis, ↑ p53, ↑ cytochrome c release, ↑ caspase-3, arrested cell-cycle at G2/M phase	
EQNPs	87.3 ± 0.78 nm	NPs synthesized by CS-MA-TPGS polymer and hot homogenization method	A549 and NCI-H460 lung cancer cells	↑ Cellular uptake, ↓ P-gp, ↓ nEGFR, ↑ apoptosis	Ganthala et al. (2022)
Ch-HNT-g-C3N4-Q NPs	Average particle size: 454.65 nm	NPs prepared by stirring process (ultrasonic bath)	MCF-7 breast cancer cells	↑ Cytotoxicity, ↑ apoptosis	Sabzini et al. (2022)
Q-F NPs	160 ± 25 nm	NPs prepared by dissolution technique	DC2.4 dendritic cells, B16F10 melanoma, and 4T1 mouse breast cancer cells; male C57BL/6 mice and Balb/c mice inoculated with B16F10 cells	*In vitro:* ↑ Photothermal therapy, modulating the tumor immunosuppressive microenvironment, cancer cell destruction, tumor antigen release, ↑ dendritic cell maturation, ↑ T cells activation, ↓ PD-L1	Li et al. (2022)
				*In vivo:* ↑ survival time, potent anti-tumor immune memory to control tumor metastasis and recurrence	
ZnO-Q NPs	Average size: 20–25 nm	NPs prepared by dissolution technique	PA-1 human ovarian cancer cells	↑ Intercellular oxidative stress, depolarization of the mitochondrial membrane, ↑ late apoptosis, activation of intrinsic apoptosis signaling pathway	Ramalingam et al. (2022)
PLGA-EGCG-NPs	175.8 ± 3.8 nm in size	NPs synthesized by the oil-in-water emulsion solvent evaporation technique	A549 and H1299 lung cancer cells and patient-derived xenograft model (male NOD/SCID mice)	*In vitro*: ↓ proliferation, ↑ apoptosis, ↓ NF-κB, ↓ C-MYC, ↓ Cyclin D1, ↓ Bcl-2, ↓ Bcl-xL, ↓ COX-2, ↓ TNF-α, ↓ TWIST1, ↓ MMP2	Zhang et al. (2020)
				*In vivo:* ↓ tumor volume, ↓ tumor weight, ↓ Ki67, ↓ phospho-NF-κB	
Ch-G-EGCG NPs	Average size: 141 ± 21 nm	NPs prepared by dissolution technique	HGC‐27 and MKN‐45 gastric cancer cells; HGC‐27/R or MKN‐45/R cells xenograft model (BALB/c female nude mice)	Systemic si‐TMEM44‐AS1 delivery, reverse 5‐FU resistance, ↓ cell viability, ↑ apototsis, ↑ P53 signaling pathway	Zhou et al. (2022)
Sb-AuNPs	AuNPs: 107 ± 9 nm, silibinin GNPs nanoconjugates: 163 ± 5 nm	AuNPs synthesized by trisodium citrate dihydrate (reducing agent) and subsequently conjugation with silibinin	A549 lung cancer cells	↑ Cell death, long-term stability, arrest the growth of cancer cells in G1 phase	Ravi et al. (2022)
SeNPs-API	Mean diameter of 124.3 nm	Green-synthesized SeNPs-API prepared by swirling together for 24 h at room temperature	MCF-7 breast cancer cells	↓ Cell proliferation, ↓ cell viability, ↑ oxidative stress, ↑ ROS, ↑ apoptosis, ↓ Bcl-2, ↑ Bax, ↑ caspase-3, ↑ cytochrome c release, ↑ DNA damage	Al-Otaibi et al. (2022)
API-GAL-NPs	NPs size according to used method: FESEM (60–120 nm) and TEM (85–160 nm) method	API-GAL-NPs prepared by using nanoprecipitation technique	HepG2 human liver hepatocellular carcinoma cancer cells and DEN-induced hepatocellular carcinoma rat model	*In vitro*: ↑ cellular internalization, ↑ cytotoxic effects, ↑ apoptosis	Ganguly et al. (2021)
				*In vivo*: ↓ nodule formation, ↓ MMP-2, ↓ MMP-9, ↑ apoptosis, ↑ P53, ↑ Bax, ↓ Bcl-2, ↓ Bcl-xL	

**Explanatory notes**: ↑ increased; ↓ decreased.

**Abbreviations**: NPs, nanoparticles; Res-AuNPs, resveratrol-conjugated gold nanoparticles; AITC- SiQDs, allyl isothiocyanate-conjugated with silicon quantum dots; HUVECs, human umbilical vein endothelial cells; Nrf2, nuclear factor erythroid 2–related factor 2; BRB, berberine hydrochloride; DSN, diosmin; CAS MCs, casein micelles; NF-κB, nuclear factor-kappa B, TNF-α, tumor necrosis factor alpha; VEGF, vascular endothelial growth factor; COX-2, cycloxogenase-2; Ki67, proliferation marker; Asp, aspirin; UA, ursolic acid; MP-AuNPs, mango peel phytochemicals coated gold nanoparticles; MGF-AuNPs, mangiferin conjugated gold nanoparticles; FA, folic acid; BSA, bovine serum albumin; CDF, difluorinated curcumin; PTX, paclitaxel; GA, gum arabic; Cur, curcumin; SLNs, solid lipid nanoparticles; MSNs, mesoporous silica nanoparticles; MPEG, monomethyl polyethylene glycol; PLA, poly lactide; CuO-ChNPs-Q, quercetin encapsulated chitosan functionalized copper oxide nanoparticle; PCNA, proliferating cell nuclear antigen; PLGA, poly (lactic-co-glycolic acid); EGCG, epigallocatechin-3-gallate; EQNPs, erlotinib and quercetin loaded solid lipid NPs; P-gp, P-glycoprotein; nEGFR, nuclear epidermal growth factor receptor; SeNPs-apigenin, green-synthesized selenium nanoparticles using apigenin; ROS, reactive oxygen species; Bcl-2, anti-apoptotic gene; API-GAL-NPs, galactose-tailored PLGA NPs, loaded with apigenin; DEN, diethylnitrosamine; MMP, matrix metalloproteinase; Res-AuNPs, resveratrol-gold nanoparticles; Cur-loaded PGS-NPs, curcumin-loaded nanoparticles of Poly (Glycerol Sebacate); Ch-HNT-g-C3N4-Q NPs, chitosan-halloysite-graphitic-carbon nitride-quercetin nanoparticles; Q-F NPs, quercetin-ferrum nanoparticles; ZnO-Q NPs, wurtzite-type zinc oxide-quercetin nanoparticles; 5‐FU, 5‐fluorouracil; Ch-G-EGCG, chitosan-gelatin-epigallocatechin-3-gallate; Sb-AuNPs, silibinin-conjugated gold nanoparticles.

### 3.4 Phytochemical-based nanodrugs targeting CSCs

CSCs, a subgroup of cells within the tumor, often cause tumors to recur and progress, consequently contributing to cancer cells’ migration and metastasis. CSCs are also associated with heterogeneously demonstrated resistance (Lathia et al., 2020; Babaei et al., 2021). To overcome the drug resistance of CSCs, combining two or more chemotherapeutic agents or multiple treatment modalities represents the potential anticancer strategy. One of the main strategies for overcoming or eliminating the resistance of CSCs to several drugs is represented by NPs-based drugs (Wang et al., 2015; Wang and He, 2018). The below studies focused on specifically designed phytochemical-based nanodrugs as promising tools against CSCs.

ALDH enzyme that converts aldehydes into carboxylic acids is highly expressed in hematopoietic stem and progenitor cells. Curcumin-loaded chitosan-PLGA-NPs modified with sialic acid and with anti-aldehyde dehydrogenase (Cur-Ch-PLGA-SA-anti-ALDH NPs) revealed anticancer potential against the proliferation of glioblastoma cells and brain CSCs. Interestingly, sialic acid on the surface of NPs helped permeate the blood-brain barrier using N-acetylglucosamine in human brain CSCs and U87MG glioblastoma cells (Kuo et al., 2019). Furthermore, CD123 is expressed explicitly in leukemic CSCs. Specifically designed NPs anti-CD123-Curcumin NPs (anti-CD123-Cur-NPs) increased cellular uptake and induced higher apoptosis in KG-1a human acute myeloid leukemia cells when compared with Cur-NPs, suggesting that anti-CD123-Cur-NPs successfully targeted leukemic CSCs (Nirachonkul et al., 2021). Impressively, a co-delivery system consisting of hyaluronic acid lipoid on the surface of hydrophobic PLGA NPs with paclitaxel as a chemotherapy agent and curcumin as the selective inhibitor of CSCs (HA-PLGA-PTX-Cur NPs) targeted breast CSCs through the interaction between hyaluronic acid lipid and the CD44 receptor on the membrane of breast CSCs leading to anticancer effects *via* reduced breast CSC population and inhibited their mammosphere formation and migration. Moreover, treatment with mentioned co-delivery system reduced the expression of ALDH1 in MCF7 mammospheres. In MCF7 mice xenografts, the co-delivery system enhanced anticancer efficacy through synergistic inhibition of the growth of non-breast CSCs and breast CSCs (Yang et al., 2017). Another study showed that curcumin combined with glucose nanogold particles (Cur-Glu-AuNPs) reduced radiotherapy resistance in targeted breast CSCs. In MCF-7 and MDA-MB-231 mammospheres, treatment with Cur-Glu-AuNPs was also associated with induced apoptosis followed by G0/G1 phase cell cycle arrest, increased ROS level, and reduced hypoxia-inducible factor-1 alpha (HIF-1α) and heat shock protein 90 (HSP90) expressions (Yang et al., 2020). Furthermore, 3,4-difluorobenzylidene curcumin loaded hyaluronic acid-copoly (styrene maleic acid) (DFBCur-HA-SMA) nanomicelles demonstrated anticancer properties against MiaPaCa-2 and AsPC-1 human pancreatic cancer cells. Treatment improved cellular internalization of nanomicelles in CD44+/CD133+/EpCAM + pancreatic CSCs compared to CD44-/CD133-/EpCAM- CSCs. Moreover, DFBCur-HA-SMA nanomicelles reduced the expression of CD44 and NF-κB, leading to anti-proliferative and anti-invasive effects (Kesharwani et al., 2015). Moreover, curcumin combined with naringenin loaded dextran-coated magnetic nanoparticles (Cur-Nar-D-MNPs) inhibited cell proliferation and induced apoptosis through ROS production, increased P53 and P21, and decreased TNFα and CD44 in MCF-7 human breast cancer cells. Furthermore, CUR-NAR-D-MNPs reduced the tumor volume and caused the cell cycle arrest in DMBA-induced mammary tumor in rats (Askar et al., 2021). Additionally, GANT61, a hexahydro pyrimidine derivative, can target CSCs of different types of human cancers through the GLI1 protein of the Hedgehog pathway. Encapsulated GANT61 and curcumin in PLGA NPs (GANT1-Cur-PLGA NPs) reduced cell viability, proliferation, induced autophagy by the formation of autophagosomes and autophagic flux, and triggered apoptosis in MCF-7 breast adenocarcinoma cell line. Treatment also reduced the nuclear expression of GLI1 and EGFR expression on the cellular membrane, cytoplasm, and the nucleus. Moreover, GANT1-Cur-PLGA NPs inhibited the downstream target proteins Bmi-1 and PI3K of Hedgehog and EGFR pathways (Borah et al., 2020).

Resveratrol NPs decreased metastatic markers CD133, ALDH1, CXCR4 in CSCs-enriched oral cancer cells leading to a reduction in the invasion, proliferation, and growth of CSCs. Moreover, a detailed study on fertilized chick embryos and mice xenografts confirmed that Resveratrol NPs depleted nitric oxide production and decreased angiogenesis and metastasis (Pradhan et al., 2021).

The combination of docetaxel- and sulforaphane-loaded PLGA-hyaluronic acid based NPs (DTX-SFN-PLGA-HA NPs) inhibited breast CSCs through decreased expression of cyclin D1 and β-catenin in MCF-7 breast cancer cells but was less effective in MCF-7 mammospheres with an epithelial-specific antigen (ESA)+CD44^+^CD24^−^ phenotype. Moreover, the treatment exerted substantial anticancer effects by inhibiting the self-renewal ability of breast CSCs in MCF-7 mice xenografts (Huang et al., 2016). Another study showed that sulforaphane-loaded the mineralized hyaluronic acid-SS-tetradecyl NPs (SFN/M-HA-SS-TA) inhibited breast CSCs through their specific CD44^+^ targeting and reduced CD44 and CD133 expression, expression of polycomb complex protein involved in the self-renewal of breast CSCs (Bmi1), and breast CSC-like properties, including tumor growth, invasiveness, and self-renewal in MDA-MB-231, Hs578t, and MCF7 cells and MDA-MB-231 mice xenografts (Gu et al., 2021).

Nanoliposomal quercetin combined with CD133 antiserum targeted CD44 and CD133 and decreased expression of NF-κBp65, histone deacetylase 1 (HDAC1), and cyclin D1, increased the expression of caspase-3 and E-cadherin in Eca109/9706 esophageal carcinoma cells (Zheng et al., 2014).

Encapsulated icariin and curcumin in polymer oligomeric hyaluronic acid-hydrazone bond-folic acid-biotin micelles targeted MCF-7 cells and breast CSCs through CD44, folic acid, and/or biotin and inhibited cancer cell invasion (Liu et al., 2019).

Sun et al. (2015) showed that all-trans-retinoic acid and doxorubicin NPs (ATRA-DOX-NPs) could simultaneously deliver the drug to both non-CSCs and breast CSCs to differentiate. ATRA NPs caused CSCs to differentiate into non-CSCs through reduced self-renewal capacity. Treatment also increased sensitivity to chemotherapy (DOX NPs). Therefore, combination therapy consisting of ATRA-DOX-NPs enhanced anticancer properties. NPs increased ATRA and DOX cellular uptake in ALDH^hi^ population MDA-MB-231 mammosphere cells and inhibited the cancer-initiating activity of CSCs. Moreover, ATRA-DOX-NPs decreased the expression of stemness-associated genes *Nanog*, *Sox2,* and *Oct4*.

**FIGURE 4:**
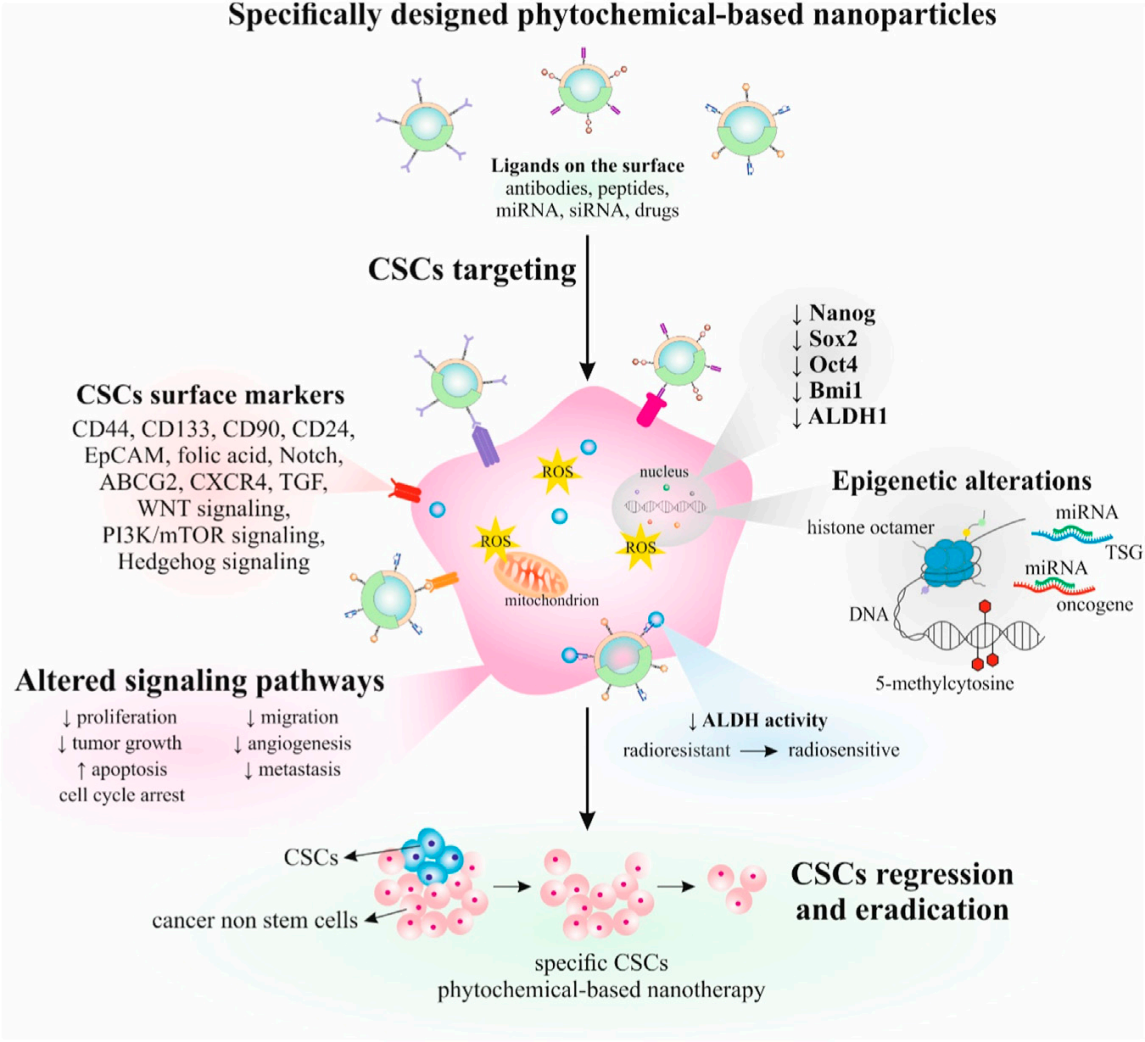
Targeting CSCs by phytochemical-based nanodrugs. Abbreviations: CSCs, cancer stem cells, TSG, tumor suppressor gene, ALDH1, aldehyde dehydrogenase 1, ↓ decreased/reduced, ↑, increased/enhanced.

Table 3 summarizes the preclinical evidence of phytochemical-based nanodrugs with potential targeting CSCs. Several studies revealedthe anticancer properties of phytochemical-based nanodrugs; however, only limited studies described their potential in the specific targeting of CSCs. Therefore, a more detailed molecular analysis of their anticancer effects, including CSCs, should be used that can reverse resistance to therapy or prevent migration and metastasis. Figure 4 illustrated the role of phytochemical-based nanodrugs in targeting CSCs.

**TABLE 3:** Phytochemical-based nanodrugs targeting CSCs.

Phytochemical-based nanodrug	Study details	Anticancer effects	Targeting CSCs	References
Cur-Ch-PLGA-SA-anti-ALDH NPs	Human brain CSCs and U87MG glioblastoma cells	↓ Proliferation, ↑ permeation of the blood-brain barrier	ALDH, brain CSCs	Kuo et al. (2019)
Anti-CD123-Cur-NPs	KG-1a human acute myeloid leukemia cells	↑ Cellular uptake, ↑ apoptosis	CD123, leukemic CSCs	Nirachonkul et al. (2021)
Co-delivery system of HA-PLGA-PTX-Cur NPs	MCF-7 mammospheres and Balb/c nude mice bearing MCF7 tumors	↓ Breast CSC population, ↓ mammosphere formation, ↓ migration, ↓ growth	CD44, ALDH1, breast CSCs	Yang et al. (2017)
			*In vivo:* ↓ growth non-breast CSCs and breast CSCs	
Cur-Glu-AuNPs	MCF-7 and MDA-MB-231 mammospheres	↑ Apoptosis, G0/G1 phase cell cycle arrest, ↑ ROS, ↓ HIF-1α, ↓ HSP90	↓ Radiotherapy resistance of breast CSCs	Yang et al. (2020)
DFBCur-HA-SMA nanomicelles	MiaPaCa-2 and AsPC-1 human pancreatic cancer cells	↑ Cellular internalization, ↓ proliferation, ↓ invasiveness, ↓ CD44, ↓ NF-κB	↓ CD44, CD133, EpCAM	Kesharwani et al. (2015)
Cur-Nar-D-MNPs	MCF-7 human breast cancer cells and DMBA-induced mammary tumor in rats	↓ proliferation, ↑ apoptosis, ↑ ROS, ↑ P53, ↑ P21, ↓ TNFα, ↓ tumor volume, cell cycle arrest	↓ CD44	Askar et al. (2021)
GANT1-Cur-PLGA NPs	MCF-7 breast adenocarcinoma cell line	↓ cell viability, ↓ proliferation, ↑ autophagy, ↑ formation of autophagosomes and autophagic flux, ↑ apoptosis	↓ GLI1, ↓ EGFR, ↓ Bmi1, ↓ PI3K, (Hedgehog signaling and EGFR pathways)	Borah et al. (2020)
Resveratrol-NPs	H-357 oral cancer cells, fertilized chick embryo, and female Balb/c mice xenografts	↓ Invasion, ↓ proliferation, ↓ growth, ↓ angiogenesis, ↓ metastasis, ↓ nitric oxide production	↓ CD133, ↓ ALDH1, ↓ CXCR4	Pradhan et al. (2021)
DTX-SFN-PLGA-HA NPs	MCF-7 breast cancer cells and mammospheres, MCF-7 female Balb/c nude mice xenografts	↓ Self-renewal ability, ↑ cellular uptake, ↓ cyclin D1, ↓ β-catenin	↓ Breast CSCs, ESA, CD44	Huang et al. (2016)
SFN/M-HA-SS-TA	MDA-MB-231, Hs578t, and MCF7 breast cancer cells, MDA-MB-231 Balb/C nude mice xenografts	↓ tumor growth, ↓ invasiveness, ↓ self-renewal	Breast CSCs, ↓ CD44, ↓ CD133, ↓ Bmi1	Gu et al. (2021)
Quercetin-anti-CD133	Eca109/9706 esophageal carcinoma cells	↓ NF-κBp65, ↓ HDAC1, ↓ cyclin D1, ↑ caspase-3, ↑ E-cadherin	CD44, CD133	Zheng et al. (2014)
ICA-Cur-Bio-oHA-Hyd-FA micelles	MCF-7 cells and breast CSCs	↑ Cellular uptake, ↓ invasion, targeted through CD44, FA and biotin	breast CSCs, CD44	Liu et al. (2019)
ATRA-DOX-NPs	ALDH^hi^ population MDA-MB-231 mammosphere cells	Simultaneous delivery to non-CSCs and breast CSCs, ↑ cellular uptake ↑ CSCs differentiation, ↓ self-renewal capacity, ↑ sensitivity to chemotherapy ↓ *Nanog*, ↓ *Sox2,* ↓ *Oct4,* ↓ cancer initiating activity of CSCs.	Breast CSCs, ALDH	Sun et al. (2015)

**Explanatory notes**: ↑ increased; ↓ decreased.

**Abbreviations**: CSCs, cancer stem cells; SA, sialic acid; ALDH, aldehyde dehydrogenase; PLGA, poly (lactic-co-glycolic acid); Cur, curcumin; HA, hyaluronic acid; PTX, paclitaxel; Cur-Glu-AuNPs, curcumin combined with glucose nanogold particles; ROS, reactive oxygen species; HIF-1α, hypoxia-inducible factor-1, alpha; HSP90, heat shock protein 90; ESA, epithelial specific antigen; DTX, docetaxel; SFN, sulforaphane; M-HA-SS-TA, mineralized hyaluronic acid-SS-tetradecyl; Bmi1, polycomb complex protein; HDAC1, histone deacetylase 1; NF-κB, nuclear factor-kappa B; ICA, icariin; Bio-oHA-Hyd-FA, polymer oligomeric hyaluronic acid-hydrazone bond-folic acid-biotin; DFBCur, 3,4-difluorobenzylidene curcumin; SMA, copoly (styrene maleic acid); ATRA, all-trans-retinoic acid; DOX, doxorubicin; Cur-Nar-D-MNPs, curcumin combined with naringenin loaded dextran-coated magnetic nanoparticles; GANT1-Cur-PLGA NPs, encapsulated GANT61 and curcumin in poly (lactic-co-glycolic acid) nanoparticles; TNFα, tumor necrosis factor alpha; GLI1, GLI, family zinc finger 1; EGFR, epidermal growth factor receptor; PI3K, phosphoinositide 3-kinase.

## 4 Benefits or risks of nanomedicine

In cancer therapies by various NPs, it is crucial to evaluate their safety, potential accumulation in non-targeted sites, clearance, excretion from the body, and others that can potentially lead to life-threatening complications (De Jong and Borm, 2008; Vinluan and Zheng, 2015).

Nanotechnology influences pharmacokinetics that can improve phytochemicals’ stability and solubility and enhance their cellular uptake at the targeted site (Vimala and Kannan, 2021). Nanotechnology also offers specific drug delivery in cancer treatment that can help overcome limitations or side effects of current cancer therapies and reduce multidrug resistance, consequently improving patients’ quality of life and survival (Gavas et al., 2021). Furthermore, phytochemical-based nanodrugs can overcome the chemotherapeutic resistance of CSCs or have the ability to resensitize them to therapy (Shen et al., 2021). Specifically designed NPs deliver drugs into cancer cells and release them only at targeted sites without damaging healthy tissues around the tumor. Moreover, this precise targeting can enhance the therapeutic efficacy of drugs in cancer cells (Mitchell et al., 2021). Furthermore, the enhanced therapeutic efficacy of NPs in cancer treatment can be achieved by hyperthermia, magnetic hyperthermia, or light-mediated phototermia (Chatterjee et al., 2011; Li et al., 2019; Vilas-Boas et al., 2020). Additionally, green nanotechnology (phytoformulations), also known as green or eco-friendly technology, can help reduce energy and fuel use to contribute to environmental sustainability without harming the environment or human health (Verma et al., 2019).

Despite mentioned beneficial properties of NPs, the carrier systems can impose risks to the patients (De Jong and Borm, 2008). Due to the ability to pass some biological barriers, NPs can exert life-threatening toxic effects, especially on essential organs, including the brain, liver, kidney, and others. For example, the accumulation of NPs in reproductive organs can damage the testis, epididymis, ovary, and uterus cells, subsequently leading to reproductive organ dysfunction (Wang et al., 2018). Among other limitations of NPs is clearance by the immune systems or impaired diffusion in the tissue microenvironment (Busatto et al., 2019). Another limitation can be the excretion of NPs from the body. Some NPs cannot be excreted and remain in the organism; however, it usually remains unclear how long NPs remain in the body and what can cause their long-term action (Fischer and ChanNanotoxicity, 2007).

In conclusion, it is crucial to evaluate and realize whether the beneficial properties or risks of using NPs predominate and which are more beneficial to the patient’s treatment.

## 5 Conclusion and outlook in the framework of 3P medicine

Phytochemicals isolated from plants demonstrate the huge potential for nanomedical applications in oncology thanks to their antioxidant, anti-inflammatory, anti-proliferative, and other health benefits. Phytochemical-based NPs can enhance anticancer therapeutic effects, improve cellular uptake of therapeutic agents, and mitigate the side effects of toxic anticancer treatments. Per evidence, phytochemical-based NPs can specifically target CSCs decreasing risks of tumor relapse and metastatic disease manifestation.

A particular value of phytochemical-based nanodrugs’ implementation is considered in the framework 3PM. The authors have presented related concepts in a series of publications (Kubatka et al., 2021; Link et al., 2021; Liskova et al., 2021; Mazurakova et al., 2022a). Personalized medicine could represent a promising way in cancer therapy through the achievement of the most effective treatment to the individual patient (Yaari et al., 2016; Mitchell et al., 2021). NPs are predicted to be the future of cancer diagnostics, medical imaging, and precise drug delivery; however, it is still important to improve their efficacy or minimize their toxicity and side effects (Li et al., 2021b). Furthermore, several studies also focused on nanotechnological cancer research aimed to cancer prevention (Zhang et al., 2016; Khan et al., 2021; Neelakandan et al., 2022), prediction (Gobbo et al., 2015; Norouzi, 2020; Jeon et al., 2022; Sousa-Junior et al., 2022), or personalized medicine (Benedetto et al., 2015; Yaari et al., 2016). The key points of 3PM are healthcare cost-efficacy and affected individuals’ life quality (Ellinger et al., 2022). Targeted treatment has to be adapted to the individualized patient profile in primary (protection against initial cancer development), secondary (protection against potential metastatic disease development), and tertiary care (towards cascading complications) (Golubnitschaja et al., 2021; Ellinger et al., 2022). To this end, advanced primary care of sub-optimal health conditions plays a pivotal role in protecting affected individuals from the heath-to-disease transition (Wang et al., 2021); principles of 3PM medicine have been recognized by WHO as an advanced approach in the area (Wang et al., 2021). Therefore, specific designed NPs tailored to patients can represent preventive and therapeutic potential in cancer management.

Except for nuclear gene mutations, mitochondria can play also a pivotal role in cancer development and progression. Mitochondria control wide range of cellular functions, including proliferation, apoptosis, signaling events, and cell homeostasis. Alterations in mtDNA copy number, mitochondrial enzymatic activities, or bioenergetic pathways are connected to worse mitochondrial health (Koklesova et al., 2022). Phytochemical-based nanodrugs can be promising agents for the maintenance of mitochondrial health and mitigation of mitochondrial impairments in innovative biomedical research and healthcare (Milane et al., 2015; Tan et al., 2019; Ashrafizadeh et al., 2020; Koklesova et al., 2021; Mazurakova et al., 2022b; Chen et al., 2022; Koklesova et al., 2022).
